# Role of the End-Point Mediators of Sympathoadrenal and Sympathoneural Stress Axes in the Pathogenesis of Experimental Autoimmune Encephalomyelitis and Multiple Sclerosis

**DOI:** 10.3389/fendo.2019.00921

**Published:** 2020-01-14

**Authors:** Ivan Pilipović, Zorica Stojić-Vukanić, Ivana Prijić, Gordana Leposavić

**Affiliations:** ^1^Branislav Jankovic Immunology Research Centre, Institute of Virology, Torlak Vaccines and Sera, Belgrade, Serbia; ^2^Department of Microbiology and Immunology, Faculty of Pharmacy, University of Belgrade, Belgrade, Serbia; ^3^Department of Pathobiology, Faculty of Pharmacy, University of Belgrade, Belgrade, Serbia

**Keywords:** sympathoadrenal system, noradrenaline, β-adrenoceptor, α-adrenoceptor, experimental autoimmune encephalomyelitis, multiple sclerosis

## Abstract

The role of stress effector systems in the initiation and progression of multiple sclerosis (MS) and experimental autoimmune encephalomyelitis (EAE), the most commonly used experimental model of MS, has strongly been suggested. To corroborate this notion, alterations in activity of the sympathoadrenal and sympathoneural axes of sympathoadrenal system (a major communication pathway between the central nervous system and the immune system), mirrored in altered release of their end-point mediators (adrenaline and noradrenaline, respectively), are shown to precede (in MS) and/or occur during development of MS and EAE in response to immune cell activation (in early phase of disease) and disease-related damage of sympathoadrenal system neurons and their projections (in late phase of disease). To add to the complexity, innate immunity cells and T-lymphocytes synthesize noradrenaline that may be implicated in a local autocrine/paracrine self-amplifying feed-forward loop to enhance myeloid-cell synthesis of proinflammatory cytokines and inflammatory injury. Furthermore, experimental manipulations targeting noradrenaline/adrenaline action are shown to influence clinical outcome of EAE, in a disease phase-specific manner. This is partly related to the fact that virtually all types of cells involved in the instigation and progression of autoimmune inflammation and target tissue damage in EAE/MS express functional adrenoceptors. Although catecholamines exert majority of immunomodulatory effects through β_2_-adrenoceptor, a role for α-adrenoceptors in EAE pathogenesis has also been indicated. In this review, we summarize all aforementioned aspects of immunopathogenetic action of catecholamines in EAE/MS as possibly important for designing new strategies targeting their action to prevent/mitigate autoimmune neuroinflammation and tissue damage.

## Introduction

Multiple sclerosis (MS) is one of the most common neurological disorders and cause of disability of young adults (1–3). Patogenetically, MS is the prototype of the autoimmune inflammatory diseases of the central nervous system (CNS), and is characterized by breakdown of the blood-brain barrier (BBB), neuroinflammation, and axonal damage (4, 5). Its pathogenesis is largely deciphered using experimental autoimmune encephalomyelitis (EAE), a group of neuroantigen-induced animal diseases (4). These models are mainly based on neuroinflammation induced by auto-reactive T helper (Th) cells (4, 6). Upon activation in draining lymph nodes (dLNs), neuroantigen-specific Th cells synthesize IL-17, IFN-γ and/or GM-CSF, and express chemokine receptors (CCR2, CCR6) to gain access in the CNS (4, 6, 7). In the CNS, upon reactivation by resident antigen-presenting cells (APCs), Th cells activate neighboring microglia and attract peripheral cells (T-cells, B-cells, inflammatory monocytes) to perpetuate neuroinflammation and cause demyelination (4, 7). With EAE development, apart from CD4+Foxp3+ regulatory T-cells (Tregs) (8), activated microglia may assume regulatory functions (through phagocytosis, anti-inflammatory mediator and growth factor release) to limit the CNS damage and promote recovery (9). Noteworthy, so far, no single experimental model covers the entire spectrum of MS immunopathological features (particularly role of CD8+ T-cells and B-cells in propagating inflammation and tissue damage in established MS), so the relevance of results from EAE models has to be critically validated (5).

MS is multifactorial disease involving genetic traits and non-genetic triggers (1, 2). Generally, physical and psychological stressors are important triggers of MS (10–12). To corroborate this notion, war veterans with stress-related disorders (associated with low levels of morning cortisol and elevated levels of noradrenaline), were found to exhibit the higher risk of being diagnosed with MS compared to those without any psychiatric disorders (13). However, not only does stress contribute to MS development, but the disease itself causes stress, creating a vicious cycle (10, 12, 14). Additionally, stress contributes to exacerbations of MS (12, 15). The pathogenetic role of stress has been ascribed not only to action of glucocorticoids, end-point mediators of hypothalamo-pituitary-adrenal system (16, 17), but also to catecholamines, end-point mediators of sympathoadrenal system consisting of sympathoneural (the key end-point mediator noradrenaline) and sympathoadrenal (the key end-point mediator adrenaline) axes (15, 16, 18). This review focuses the role of catecholamines in development of EAE/MS.

In MS, aside from sensory, motor and cognitive impairments, autonomic dysfunction (mirrored in fatigue, bladder, bowel, cardiovascular, and sexual disorders) considerably contributes to disability (19–21). It has been speculated that (i) altered sympathoadrenal system activity induced by various stressors, including the disease itself (in early phase and at the onset of exacerbations), and (ii) damage of central sympathoadrenal system neurons and their projections with the disease progression (12, 22–24) is not only consequence, but also mechanism involved in MS pathogenesis.

The sympathoadrenal system, a major communication pathway between the CNS and the immune system (25), originates from locus coeruleus (LC) (18, 26). Activation of LC leads to (i) central effects reflecting noradrenaline release (primarily via non-junctional varicosities to enable its action on non-neural cells) throughout the brain and spinal cord (SC), and (ii) peripheral effects due to release of catecholamines from adrenal medulla and sympathetic nerve fibers (18, 27–30). On the other hand, activation of peripheral immune cells activates sympathoadrenal system to secure control of the ongoing response (18, 31). Namely, cytokines released upon their activation signal to the sympathoadrenal system by stimulating proinflammatory mediator release from the CNS resident cells or by activation of afferent signaling pathways (32–36). In inflammatory autoimmune diseases, this activational effect is suggested to be superimposed on elevated sympathoadrenal system activity due to chronic stress and/or stressful adverse life events leading to its hyperactivity and proinflammatory action (31). In MS, the release of proinflammatory cytokines from activated immune cells in the CNS also contributes to sympathoadrenal hyperactivity (32, 33, 36). Stress-induced sympathoadrenal activation prior to these diseases is suggested to induce low grade self-perpetuating lymphoid tissue and systemic inflammation that further increases sympathoadrenal activity and alters immune system reactivity to enable autoreactive lymphocyte activation (31). This, in return, contributes to sympathoadrenal activation and the promotion of inflammation (31, 37). Adding to the complexity, innate and adaptive “catecholaminergic” immune cells also synthesize catecholamines (29, 30, 38) to regulate inflammatory/immune responses (39). However, in inflammatory autoimmune diseases, they may form an alternative catecholamine source with role in promotion of inflammation (40–43). Namely, activated immune cell-derived catecholamines are suggested to drive an autocrine/paracrine self-amplifying feed-forward loop to increase synthesis of proinflammatory cytokines in myeloid cells (41, 44). Thus, in early phases of the diseases neurocrine/endocrine- and autocrine/paracrine-derived catecholamines may synergistically act to promote inflammation.

Catecholamines exert immunomodulatory effects through β- and α-adrenoceptors expressed on almost all types of immune cells, but majority of their effects are β_2_-adrenoceptor-mediated (18, 25, 43, 45–60). Monocytes/macrophages, together with dendritic cells, constitute the mononuclear phagocyte system, which plays a key role in maintaining tissue integrity, its restoration after injury, and the initiation, direction and resolution of innate and adaptive immunity. Catecholamines modulate their activity in a context-dependent manner, so they exert both proinflammatory (61–63) and anti-inflammatory (64, 65) effects depending on a number of factors (25), including adrenoceptor subtype (66, 67), adrenoceptor agonist concentrations (68), and the timing of adrenoceptor engagement in relation to antigen stimulation (69). Thus, it seems obvious that their action in EAE/MS has to be disease phase-dependent. In this review, considering EAE/MS pathogenesis, catecholamine influence on microglia and Th17/Treg axis is focused. Several stress paradigms induce β-adrenoceptor antagonist (propranolol) preventable microglial activation and synthesis of inflammatory mediators exaggerating proinflammatory responses to subsequent immunological stimuli (70). β_2_-adrenoceptor activation in lipopolysaccharide-stimulated dendritic cells diminishes IL-12 secretion, leading to a shift in the IL-12/IL-23 ratio and thereby promotes the generation of CD4+ T cells that produce lower amounts of IFN-γ (Th1 signature cytokine) and higher levels of IL-17 (Th17 signature cytokine) (71).

## Central Noradrenaline in Pathogenesis of EAE/MS

### Human Data

It has been shown that in MS noradrenaline levels decrease in the tissue surrounding LC (72) reflecting the disease-induced neuronal damage in LC (72). At present, there is no data on effects of the disease on the other “descending catecholaminergic system neurons” projecting to the spinal cord. On the other hand, several studies showed that cerebrospinal fluid levels of noradrenaline metabolite 3-methoxy-4-hydroxyphenylglycol, a marker of central noradrenergic activity, do not change in MS (73, 74). Given that peripheral and central administration of cytokines to rodents increased noradrenaline synthesis and 3-methoxy-4-hydroxyphenylglycol levels in brain (75), it may be assumed that elevated noradrenaline synthesis and turnover in non-damaged brain structures overcame diminished noradrenaline synthesis in those affected by the disease. On the other hand, combination of lofepramine or maprotiline (noradrenaline reuptake inhibitors) with levodopa (after conversion to dopamine metabolizes to noradrenaline) exhibited therapeutic effects in MS (76). However, given that dopamine itself exerts beneficial effects on the disease (77), these effects cannot be ascribed to the rise in the central noradrenaline level. On the other hand, although combined treatment with lofepramine and phenylalanine (upstream noradrenaline precursor) was initially shown to moderate clinical symptoms of MS (78), follow-up rigorously controlled study put the benefits of this therapy into question (79). Additionally, there are limited and inconsistent data on the therapeutic effects of β_2_-adrenoceptor agonists, such as salbutamol (albuterol), in MS. Namely, depending on type and phase of MS both adverse and benefitial effects have been described (80–82). To potentiate need for further studies on role of central noradrenaline in MS, several studies provided evidence that anti-stress therapies, including exercise (83–85), mindfulness meditation (86–88), and yoga (89), moderate MS symptoms (depression, anxiety, fatigue, cognitive dysfunction). In the same line are data from a population-based study indicating that the incidence of MS was negatively associated with use of fenoterol, a β_2_-adrenoceptor agonist, but not salbutamol belonging to the same drug class (90). This was ascribed to differences in their functionality, as fenoterol differently from salbutamol significantly stimulates cyclic-adenosine monophosphate (90).

### Animal Data

The study encompassing dogs suffering from EAE showed that cerebrospinal fluid and white matter noradrenaline levels rise early after the immunization, but decrease in the clinical phase of the disease (91). Consistently, decline in SC and/or brainstem noradrenaline concentration was found at the peak of EAE in Lewis and Dark Agouti (DA) rats (45, 92–94). Noradrenaline concentration in SC also decreased with EAE progression in C57BL/6 mice (72, 95). This was attributed to disease-related damage of LC noradrenergic neurons (72) and/or axonal damage in SC (92). Additionally, it was reported that electrolytic destruction of LC noradrenergic neurons attenuates the disease in Wistar rats (96). Furthermore, central noradrenaline depletion (decrease in noradrenaline level by ~85% without changes in dopamine) by intracisternal-ventricular 6-hydroxydopamine injections reduced motor deficit in Lewis EAE rats (97, 98). Conversely, in C57BL/6 mice developing chronic EAE, treatment with N-(2-chloroethyl)-N-ethyl-2 bromobenzylamine, selective neurotoxin for rodent LC neurons, exacerbated the disease (99). This discrepancy could be related to N-(2-chloroethyl)-N-ethyl-2 bromobenzylamine–induced increase in the central extraneuronal noradrenaline level due to its inflow from non-lesioned regions, so that noradrenaline levels were reduced by only 10–30% (100). Additionally, treatment with propranolol, a non-selective β-adrenoceptor antagonist, depending on its onset relative to immunization, produced different effects on clinical outcome of EAE in rats (101, 102). Propranolol treatment starting 3 days before immunization moderated clinical and histological picture of EAE in DA rats (102), whereas the treatment beginning at immunization prolonged the disease duration in Lewis rats (101). When administered over effector phase of EAE to Lewis rats, propranolol exacerbated the disease (103), or produced no effect (101), while propranolol treatment in DA rats starting before the onset of clinical EAE decreased the disease severity (45). Given that propranolol crosses the BBB (104), the latter findings were consistent with data indicating that chemical depletion of central noradrenaline starting before the effector phase of EAE may remove an effector amplification mechanism leading to suppression of the paralysis (97). The inconsistencies in data from different propranolol studies may be associated with differences in drug dose regimen and/or treatment onset/duration, as well as animal genetic makeup, immunization protocols (possibly affecting the kinetics in development of sympathoadrenal neuron damage).

The ameliorating effect of propranolol on the clinical outcome of EAE in DA rats was linked with upregulated expression of nuclear factor (erythroid-derived 2)-like 2 (Nrf2) and heme oxygenase-1, a Nrf2-regulated gene with a crucial role in the prevention of neuroinflammation (105, 106). This partly reflected propranolol-induced upregulation of CX3CR1, the receptor for fractalkine (CX3CL1), which activates the Nrf2 signaling in microglial cells to limit their activation (107). Nrf2 recognizes an enhancer sequence termed antioxidant response element that is present in the regulatory regions of over 250 genes (108), and is implicated in the modulation of inflammation through crosstalk with the transcription factor NF-κB, the principal regulator of inflammation (109). Consistently, compared with saline-injected controls, in propranolol-treated rats the frequencies of IL-1β- and IL-23-expressing cells among microglia, and microglia expression level of IL-6 and CCL-2, the chemokine recruiting inflammatory monocytes and T-cells to the sites of inflammation (110), was decreased (45). Additionally, in accordance with role of CX3CR1 in regulation of the expression of TAM receptors (111), which are essential in apoptotic cell phagocytosis (112), so that their deficiency is linked with autoimmune disease progression (110), the frequency of phagocytic cells among microglia was significantly increased in propranolol-treated rats (45). As expected (9, 113, 114), this correlated with the increased proportion of anti-inflammatory CD163- and IL-10-expressing microglia (45). In keeping with alterations in phenotypic and functional profile of microglia, in propranolol-treated EAE rats the infiltration of SC with blood-borne inflammatory monocytes and Th cells, their reactivation/proliferation and differentiation toward highly pathogenic IL-17/IFN-γ/GM-CSF co-producing Th17 cells was impaired (45).

On the other hand, administration of prazosin, an α_1_-adrenoceptor antagonist, throughout the disease or effector phase alone suppressed active and passively transferred EAE in rats (103, 115, 116). This was related to blockade of disease-promoting α_1_-adrenoceptor–mediated vascular action (115).

### Putative Research Directions

Considering all the aforementioned, it is clear that many important issues still remain to be addressed to fully enlighten the role of sympathoadrenal system in EAE/MS pathogenesis, but to mention a few. To confirm changes in sympathoadrenal system reactivity during EAE development, noradrenaline concentration in SC along with development of sympathoadrenal neuron lesions, should be examined in distinct EAE models and distinct phases and types of MS. Additionally, considering that rodent microglia synthesize catecholamines (45), it should be investigated whether these cells, as macrophages (41), may enhance local inflammation by an autocrine/paracrine feedback mechanism. Furthermore, given that functional β_1_- and β_2_-adrenoceptors were revealed on microglia (46), further research to delineate β_1_-adrenoceptor-mediated from β_2_-adrenoceptor-mediated effects on microglia in this model is necessary. Moreover, given that microglia express α_1_-adrenoceptor (47), putative α_1_-adrenoceptor-mediated effects of catecholamines on microglia from EAE rats are also worth examining.

## Peripheral Catecholamines in Pathogenesis of EAE/MS

### Human Data

In favor of peripheral sympathoadrenal dysregulation in MS, in chronic progressive (CP) MS increase in circulating noradrenaline level was found (117). Differently, in relapsing-remitting (RR) MS its level is decreased (118). Additionally, in active RR MS, circulating levels of adrenaline and noradrenaline are lower than in stable disease (23). Alterations in lymphocyte catecholamine levels also occur in MS (119), so higher adrenaline in the first-attack MS patients and lower noradrenaline in RR MS were found (119). Higher noradrenaline level was also measured in peripheral blood mononuclear cells (PBMC) from MS patients (120). Additionally, upregulated β-adrenoceptor on T-lymphocytes from CP MS patients (121, 122), and on PBMC from RR and secondary progressive MS patients was reported (123–125). There is no data on the expression of α-adrenoceptors on peripheral immune cells from MS patients.

### Animal Data

In Lewis EAE rats, splenic noradrenaline concentration decreased during the inductive phase of the disease (126). Our recent study demonstrated reduced noradrenaline concentration in dLNs from DA rats on the 7th day post-immunization (59). However, noradrenaline content was increased in dLN cells constituting, most likely, a compensatory mechanism (42, 127, 128). Early studies in active (129) and adoptively transferred (130) Lewis rat EAE showed more severe disease in adult rats subjected to 6-hydroxydopamine–induced sympathectomy at birth. These findings should be interpreted with caution, as neonatally administered 6-hydroxydopamine crosses the BBB (129), so aside from peripheral sympathectomy, it permanently increases noradrenergic innervation in hind brain (131). Administration of isoproterenol, a non-selective β-adrenergic agonist, during preclinical phase of EAE in Lewis rats suppressed the disease severity, while propranolol did not produce any effects (101). Conversely, we showed that propranolol administration throughout preclinical phase of EAE in DA rats moderated the disease severity (132). This discrepancy could be related to recent findings indicating that isoproterenol represents a novel type of α_1A_-adrenoceptor partial agonist (133), and differences in propranolol dose, particularly as in the rat there are strain differences in its metabolism. Furthermore, it has recently been reported that increased systemic noradrenaline levels due to sympathoneural system hyperactivity (134) in mice constitutively lacking α_2a/c_-adrenoceptor (constituting an important negative-feedback mechanism required for the presynaptic control of neurotransmitter release from sympathetic fibers) is associated with diminished pathogenic T-cell responses and CNS inflammation in EAE (135). These findings might be explained by data suggesting that prolonged sympathoneural activation (as it is in late phases of inflammatory autoimmune diseases) leads to anti-inflammatory sympathoneural action (31). The moderating effect of propranolol on clinical outcome of EAE in DA rat model was ascribed to diminished CD4+ T-cell activation/proliferation and Th17 cell generation in dLNs (132), due to impaired migration of neuroantigen-carrying APCs from the site of immunization to dLNs, reflecting decreased expression of CCL19/21, chemokines driving their migration in dLNs (132). On the other hand, study on propranolol effects on dLN cells recovered in the inductive phase of EAE in the presence of arterenol (synthetic noradrenaline) or its absence showed that it enhanced CD4+ cell IL-2 synthesis and proliferation (43). Additionally, propranolol augmented differentiation of Th17 cells in dLN cell cultures by increasing RORγt expression in CD4+ cells, and production of cytokines driving/maintaining Th17 cell differentiation (IL-1β and IL-23) by APCs (43). The discrepancy between the effects of propranolol *in vivo* and *in vitro* could be reconciled by data indicating that the number of autoantigen-carrying APCs in dLNs critically determines the magnitude of the primary (auto)reactive CD4+ T-cell response and clinical outcome of autoimmune responses (136, 137).

Until recently, role of α-adrenoceptor in EAE was exclusively related to the effector phase of the disease (103, 115, 138). Our recent study showed the expression of α_1_-adrenoceptor on Tregs (but not on effector CD4+ T-cells) and APCs from dLNs of DA rats in the inductive phase of EAE (59). More important, it showed that prazosin suppressed proliferation of neuroantigen-stimulated CD4+ T-cells in dLN cell cultures, by increasing the frequency of Tregs and their Foxp3 and TGF-β expression, and decreasing co-stimulatory molecule expression on APCs (59). Moreover, prazosin also decreased IL-1β and IL-23 production in EAE rat dLN cell cultures, and consequently the generation of Th17 cells, including the most pathogenic GM-CSF–producing ones (59).

### Putative Research Directions

To fulfill composite picture of sympathoadrenal system modulation of EAE/MS development, several issues need to be resolved. In light of findings suggesting that sympathoneural changes in inflammatory autoimmune diseases may be organ-specific (31), it should be answered if sympathoadrenal influence on (auto)immune response in dLNs changes with progression of EAE, and at which time-point this change occurs, as well as to elucidate adrenoceptor types involved in immunoregulation. Furthermore, as human studies directly linking stress and (auto)immune response are lacking, owing to practical and ethical concerns (139), EAE models, despite limitations (5), should be considered for investigating role of stress in triggering MS, and particularly pharmacological treatments affecting catecholamine action trough distinct types of adrenoceptors. Considering that individual's elevated noradrenergic tone (e.g., due to genetics) may favor MS onset (140), such investigation should encompass animals of different genetic makeup.

## Conclusions

**Figure 1:**
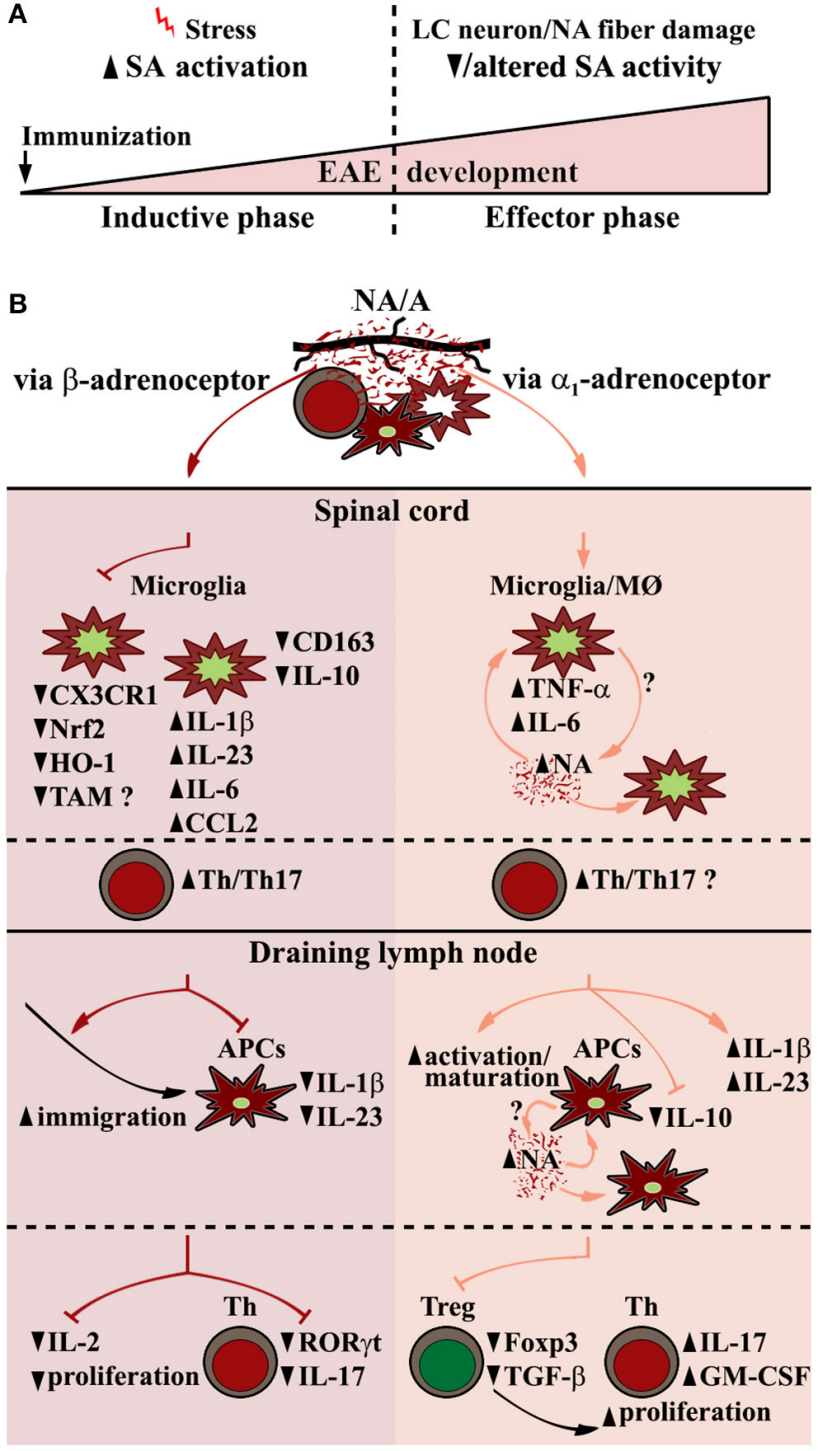
EAE-related alterations in sympathoadrenal system (SA) and putative central and peripheral effects of its key end-point mediators (noradrenaline and adrenaline) contributing to EAE pathogenesis. **(A)** Biphasic changes in SA over the course of EAE encompass SA overactivation in preclinical EAE stage (premorbid/disease-related stress), followed by its diminished and possibly qualitatively altered activity in clinical stage of the disease partly due to locus coeruleus (LC) neuron damage. **(B)** (Spinal cord) Central noradrenaline (NA)/adrenaline (A) acting through β-adrenoceptor in early phases of the disease downregulate microglial expression of nuclear factor (erythroid-derived 2)-like 2 (Nrf2), and its key anti-inflammatory downstream target genes, including those encoding heme oxygenase-1 (HO-1), and possibly TAM (Tyro3, Axl, and Mertk) receptors involved in phagocytosis via C-X3-C motif chemokine receptor 1 (CX3CR1)-dependent and CX3CR1-independent mechanisms. This leads to shift toward more proinflammatory microglial phenotype mirrored in increased expression of proinflammatory cytokines/chemokines (e.g., IL-1β, IL-23, IL-6, CCL2), followed by diminished expression of anti-inflammatory microglial markers (e.g., CD163, IL-10), and consequently, increased infiltration of spinal cord with Th cells, their reactivation/proliferation and differentiation toward pathogenic Th17 cells. (dLN, draining lymph node) NA/A acting through β-adrenoceptor enhance antigen-carrying antigen presenting cell (APC) migration to dLN, whereas impair their synthesis of Th17 polarizing cytokines (IL-1β, IL-23), and CD4+ cell expression of RORγt and their proliferation. On the other hand, α_1_-adrenoceptor–dependent stimulation leads to APC activation/maturation and augmented Th17-polarizing cytokine expression, followed by decrease in Foxp3+ Th cell (Treg) number and expression of Foxp3 and TGF-β leading to increased proliferation of Th cells and their IL-17/GM-CSF synthesis. Proinflammatory NA effects in myeloid cells, including microglia and macrophages (MØ) and peripheral APCs, may be self-amplified through a NA-α_1_-adrenoceptor loop.

Collectively, available data suggest that alterations in sympathoadrenal system activity due to premorbid/disease-induced stress and disease-associated sympathoneural damage contribute to EAE and possibly MS onset and development (affecting distinct types of immune cells, and particularly important microglia, as depicted in Figure 1), respectively. However, further research to elucidate noradrenaline/adrenaline immunomodulatory action in the target organ and lymphoid organs/blood, in distinct phases of EAE (and in distinct EAE models) and MS alike, is necessary to envisage significance of alterations in sympathoadrenal immunomodulatory action for susceptibility to/progression of EAE/MS, and consequently consider possibilities to manipulate catecholamine action to prevent/mitigate them. To emphasize significance of this research it should be pointed that adrenergic drugs are safe and cost-effective.

## Author Contributions

GL and IPi wrote the manuscript. All authors participated in data collection and interpretation, critically revised the manuscript, and approved the final version for submission.

### Conflict of Interest

The authors declare that the research was conducted in the absence of any commercial or financial relationships that could be construed as a potential conflict of interest. The reviewer DM declared a shared affiliation, with no collaboration, with the authors ZS-V and GL to the handling editor at the time of the review.
